# Outstanding Characteristics of Thai Non-GM Bred Waxy Cassava Starches Compared with Normal Cassava Starch, Waxy Cereal Starches and Stabilized Cassava Starches

**DOI:** 10.3390/plants8110447

**Published:** 2019-10-24

**Authors:** Roselawatee Toae, Klanarong Sriroth, Chareinsuk Rojanaridpiched, Vichan Vichukit, Sunee Chotineeranat, Rungtiva Wansuksri, Pathama Chatakanonda, Kuakoon Piyachomkwan

**Affiliations:** 1 Department of Biotechnology, Faculty of Agro-Industry, Kasetsart University, Bangkok 10900, Thailand; toae.roselawatee@gmail.com (R.T.); aapkrs@ku.ac.th (K.S.); 2 Department of Agronomy, Faculty of Agriculture, Kasetsart University, Bangkok 10900, Thailand; agrcsr@yahoo.com (C.R.); vichanvic@gmail.com (V.V.); 3 Cassava and Starch Technology Research Team, National Center for Genetic Engineering and Biotechnology, Pathum Thani 12120, Thailand; sunee@biotec.or.th (S.C.); rungtiva.wan@biotec.or.th (R.W.); 4 Kasetsart Agricultural and Agro-Industrial Product Improvement Institute, Kasetsart University, Bangkok 10900, Thailand

**Keywords:** waxy, cassava, non-GM, starch

## Abstract

Waxy cassava roots of nine varieties successfully developed in Thailand by a non-genetic modification (non-GM), conventional breeding method were used for extracting starches and their starch physico-chemical properties were evaluated and compared with normal cassava starches, commercial waxy starches (i.e., waxy maize starch and waxy rice starch) and commercial stabilized starches (i.e., acetylated starch and hydroxypropylated starch). All waxy cassava varieties provided starches without amylose while normal cassava starches contained 18%–20% amylose contents. As determined by a Rapid Visco Analyzer (RVA) at 5% (dry basis), waxy cassava starches had the highest peak viscosity and the lowest setback viscosity. Cooked paste of waxy cassava starches had the greatest clarity and stability among all starches during storage at 4 ℃ for 7 days as evidenced by its high light transmittance (%T) at 650 nm. No syneresis was detected in waxy cassava starch gels after subjecting to four freeze-thaw cycles (4 weeks) indicating high potential use of waxy cassava starches, free from chemicals, to replace stabilized starches as thickening and texturing agents in food products.

## 1. Introduction

Cassava (*Manihot esculenta* Crantz) is an important food crop in many parts of Asia, Africa and South America. It is mainly used as a raw material for extracting cassava starch which contains low protein, lipid and phosphorus and is considered a great source of carbohydrate. Cassava starch is pure white and its granules are generally round or oval, with a flat surface on one side (truncated end) and is approximately 7–20 µm in size [1] depending on genetics, growth periods and seasons [2]. The amylose contents of cassava starches from a world collection of 4,050 genotypes were reported between 15.2%–26.5% and 20.7% in average [3]. As compared with other crop starches including maize, rice, wheat and potato starches, cassava starch has fewer variations in its compositions. Cassava starch paste possesses unique characteristics including bland paste with higher viscosity and clarity as well as lower retrogradation rates than starches from cereals [1]. Nevertheless, there are limitations in cassava starch applications due to its properties, i.e., their swollen, gelatinized granules cannot retain granular structure and thus collapsing instantaneously [4].

Recently, various genetic cassava starches (natural mutation; non-genetic modification (non-GM) and GM containing absent and high amylose content have been reviewed [5,6,7]. In particular, a natural or spontaneous mutation with amylose-free or waxy cassava starch (WC) which is absent in granule-bound starch synthase (GBSS) enzyme for amylose biosynthesis was developed and characterized for industrial applications [1,8,9,10,11,12,13,14,15]. The natural mutation or non-GM waxy cassava, i.e., AM 206-5, was discovered by [8]. However this clone, when planted, had a low root yield and its small plant stature was not suitable for commercial purpose [11]. The International Center for Tropical Agriculture (CIAT) in collaboration with Thai Tapioca Development Institute (TTDI) and Kasetsart University, therefore, further developed new waxy cassava varieties by cross-pollinations between this low yielding waxy starch cassava mutant and normal cassava varieties with good agronomic traits, good growth form, regular root yield and good adaptability to a similar environment with that of Thailand [10,11].

The properties of non-GM WC was investigated with no change in starch granule size or shape compared with normal cassava starch (NC) [8] in contrast with the report of [14], which found that WC exhibited a larger size than NC. The pasting behavior of WC indicated its higher peak viscosity and gel breakdown but lower setback in comparison with NC [8,14]. Furthermore, solubility was reduced, while the swelling power and volume fraction of the dispersed phase were higher in WC [8]. However, it was reported that WC gels had higher solubility and swelling power than NC [14]. The paste clarity of WC was evidently higher than NC. In addition, no retrogradation or syneresis was detected in WC gels after refrigeration or freeze-thaw cycles [9,11,14]. WC also contained a lower cyanide content than NC [11]. The stability in acid, alkaline and shear of non-GM WC were relatively similar to NC [9]. Compared with commercial waxy starches such as commercial potato starch, commercial waxy maize starch (CWM) and commercial waxy rice starch (CWR), WC gels showed better characteristics including paste clarity, swelling power, solubility and freeze-thaw stability [9,12].

However, no previous report has compared the properties of WC with chemically modified stabilized starches such as acetylated starch and hydroxypropylated starch. Acetylated starches are generally prepared by esterification with acetic anhydride or vinyl acetate to replace the hydroxyl groups of native starch with acetyl groups. Hydroxypropylated starches are prepared by etherification with propylene oxide in the presence of an alkaline catalyst. Acetylated and hydroxypropylated starches had reduced retrogradation rate and textural changes during refrigerated storage [16,17,18] and thus are most commonly used for the purpose of reducing retrogradation in refrigerated food products [19], which imparts the textural shelf life of food products. This is also highly important in frozen foods as the retrogradation of starch is accelerated at cold temperatures, leading to an opaque, gelled and/or chunky texture with eventual syneresis or “weeping” of liquid from the gel [20].

The objectives of this study were, therefore, to characterize the starches from nine Thai non-GM bred waxy cassava varieties developed in Thailand and to compare them with NC, commercial waxy cereal starches and stabilized starches.

## 2. Results and Discussion

### 2.1. Amylose Contents

**Figure 1:**
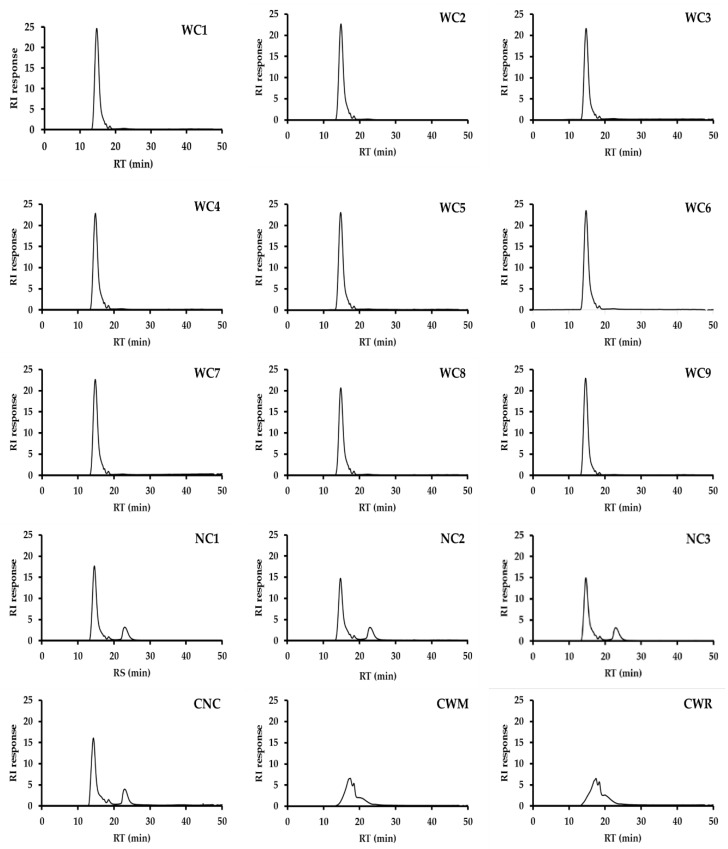
Chromatograms of nine Thai non-GM bred waxy cassava starches (WC1–WC9), three wild-type normal cassava starches (NC1–NC3), commercial normal cassava starch (CNC), commercial waxy maize starch (CWM) and commercial waxy rice starch (CWR) analyzed by high performance size exclusion chromatography (HPSEC).

The amylose contents of starch samples are shown in Table 1 and Figure 1. Waxy cassava starches (WC1–WC9), CWM and CWR contained no amylose, while the amylose contents of normal cassava starches (NC1–NC3) and commercial normal cassava starch (CNC) were in a range of 18%–21%. The amylose contents of waxy cassava starches determined in this study were in agreement with those of the spontaneous mutation WC reported by other researchers analyzed by colorimetry, high performance size exclusion chromatography (HPSEC), differential scanning calorimetry (DSC) and iodine binding capacity (IBC) methods [1,8,11,12,14].

### 2.2. Morphology and Size Distribution

**Figure 2:**
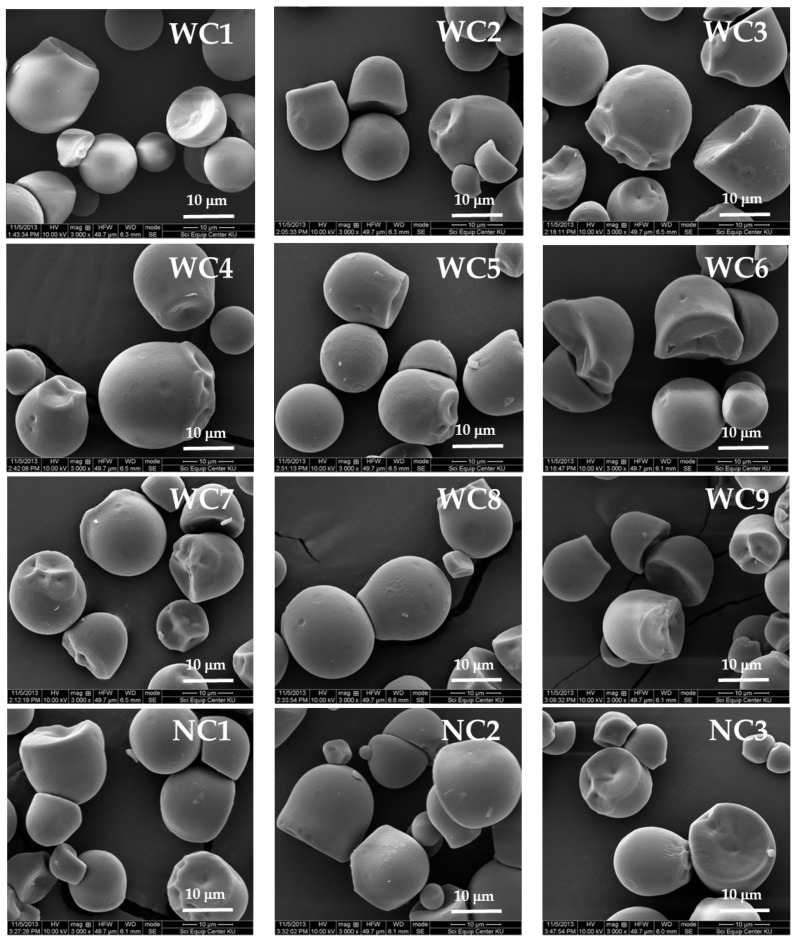
SEM photographs (x3000) of nine Thai non-GM bred waxy cassava starches (WC1–WC9) and three wild-type normal cassava starches (NC1–NC3).

**Figure 3:**
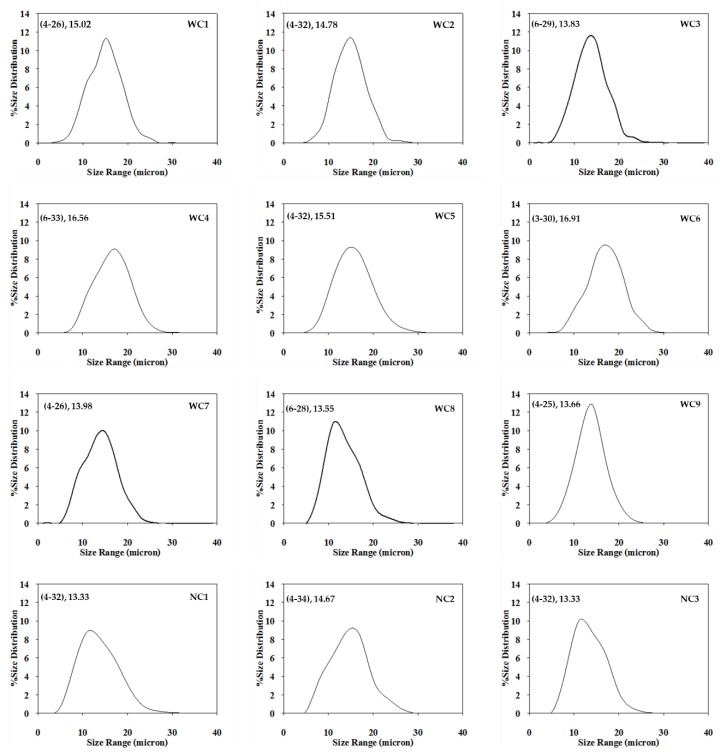
Granule size distributions of nine Thai non-GM bred waxy cassava starches (WC1–WC9) and three wild-type normal cassava starches (NC1–NC3).

SEM photographs of starch granules presented in Figure 2 confirmed that granular morphologies of waxy cassava starches (WC1–WC9) and normal cassava starches (NC1–NC3) were similar. The starch granules of both starch groups were spherical or oval with truncated ends and some of them showed facets as reported previously by [1,8,12]. The granule size distributions of waxy and normal cassava starches are shown in Figure 3. Starch granule sizes of WC1–WC9 were in the range of 3–33 µm with an average size of 13.55–16.91 µm, while those of NC1-NC3 were 13.33–14.67 µm. Starch granule sizes of waxy cassava and normal cassava were previously reported at 10–15 µm [7] and 12–17 µm for only waxy cassava [1]. However, [14] reported that waxy cassava exhibited a larger average granule size (15.9 µm) than normal cassava (14.3 µm).

### 2.3. Starch Paste Behavior

**Figure 4:**
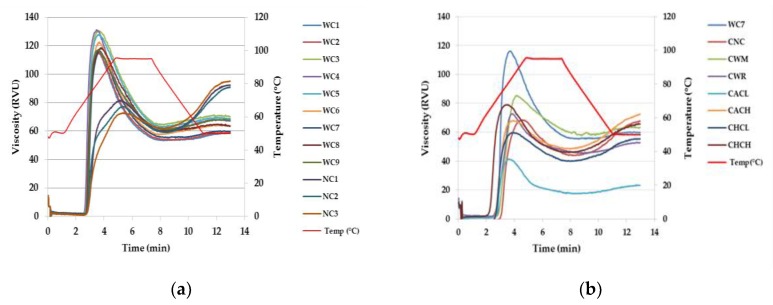
Pasting profiles of (a) nine Thai non-GM bred waxy cassava starches (WC1–WC9) and three wild-type normal cassava starches (NC1–NC3) and (b) Thai non-GM bred waxy cassava starch (WC7), commercial normal cassava starch (CNC), commercial waxy maize starch (CWM), commercial waxy rice starch (CWR) and commercial acetylated cassava starches with low and high degrees of substitutions (CACL and CACH) and commercial hydroxypropylated cassava starches with low and high degrees of molar substitutions (CHCL and CHCH).

Pasting characteristics of starch samples are shown in Table 2 and Figure 4. Pasting temperatures of waxy cassava starches (WC1–WC9) were relatively similar to normal cassava starches (NC1–NC3) (70–72 ℃ and 71–73 ℃, respectively), while other researchers reported that the pasting temperature of waxy cassava starch was slightly higher than normal cassava starch [8,9]. The pasting viscosity of WC1-WC9 (116–131 RVU) were significantly higher than that of NC1–NC3 (80–94 RVU). The breakdown viscosity of WC1–WC9 (60–70 RVU) were considerably higher than that of NC1–NC3 (29–32 RVU) indicating their lower resistance to high temperature and sensitivity to shearing stress [7], while their final viscosity showed the opposite trend (62–67 and 88–101 RVU, respectively) leading to lower values of their setback viscosity (6–10 and 31–37 RVU, respectively). 

When compared among waxy cassava starches (WC1–WC9) and commercial starches, i.e. normal cassava starch, waxy maize starch and waxy rice starch (CNC, CWM and CWR, respectively), CNC exhibited the highest pasting temperature followed by CWM, WC1–WC9 and CWR (74, 72, 70–72 and 67 ℃, respectively), while it was previously reported that pasting temperature of WC (67 ℃) was lower than CWM (71 ℃) and CWR (67 ℃) [8]. Peak viscosity of WC1–WC9 were the highest (116–131 RVU) when compared with other commercial starches (69, 85 and 73 RVU for CNC, CWM and CWR, respectively). While a breakdown viscosity of WC1–WC9 were the highest followed by CWM, CWR and CNC (60–70, 28, 28 and 25 RVU, respectively), suggesting a lower tolerance to shear stress of waxy cassava starches. After cooling, the final viscosity of waxy cassava starches were lower than that of CNC (62–67 and 68 RVU, respectively) due to the absence of amylose but slightly higher than that of CWM and CWR (63 and 53 RVU, respectively).

The degrees of substitutions of commercial acetylated cassava starches with low and high degrees of substitutions (CACL and CACH) were 0.013 and 0.077, respectively, while the degrees of molar substitution of commercial hydroxypropylated cassava starches with low and high degrees of molar substitutions (CHCL and CHCH) were 0.037 and 0.101, respectively (data not shown). According to data reported in Table 2, pasting temperatures of stabilized starches decreased when the degrees of substitutions increased, corresponding to a previous report by [21], while the increase in peak viscosity occurred because of the decrease in associative forces within the starch granules [22]. Moreover, the low breakdown viscosity and setback viscosity were observed indicating greater starch paste stability and lower retrogradation tendency of stabilized starches. These results indicated lower shear and heat resistance but a comparable paste stability of waxy cassava starches with commercial stabilized starches. 

### 2.4. Paste Clarity

Paste clarity of 2% starch pastes from waxy cassava starches (WC1–WC9) and normal cassava starches (NC1–NC3) before and after storage at 4 °C for 7 days are presented in Table 3. The paste clarity of WC1–WC9 and NC1–NC3 before storage were 91%–95% and 53%–56%, respectively, while they were 92%–95% and 28%–37%, respectively after storage at 4 ℃ for 7 days. These results showed that %T before storage of WC1–WC9 were not different and their changes after storage for 7 days (Δ%T) were similarly not detected. WC7 was chosen as a representative of all waxy cassava starches for further characterization based on its good agronomic traits and high starch content [11].

(1) $$\Delta T^{}=\frac{\left(\%T\;of\;freshly\;cooked\;sample\;at\;0\;day-\%T\;of\;sample\;stored\;for\;7\;days\right)}{\%T\;of\;freshly\;cooked\;sample\;at\;0\;day}\times 100$$

**Figure 5:**
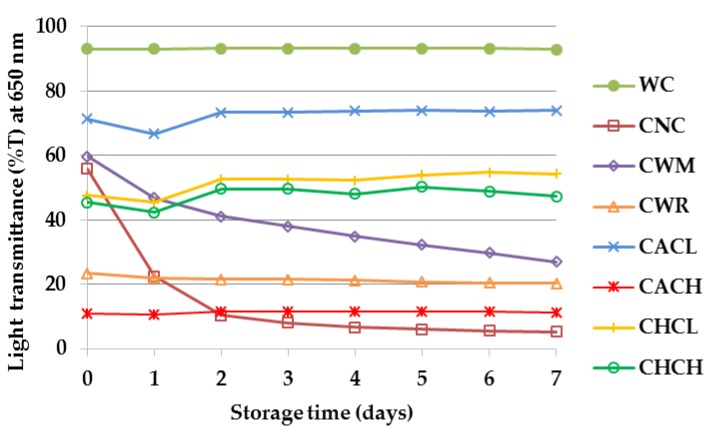
Light transmittance (%T) at 650 nm of 2% starch pastes from Thai non-GM bred waxy cassava starch (WC), commercial normal cassava starch (CNC), commercial waxy maize starch (CWM), commercial waxy rice starch (CWR) and commercial acetylated cassava starches with low and high degrees of substitutions (CACL and CACH) and commercial hydroxypropylated cassava starches with low and high degrees of molar substitutions (CHCL and CHCH) during storage at 4 ℃ for 7 days.

**Figure 6:**
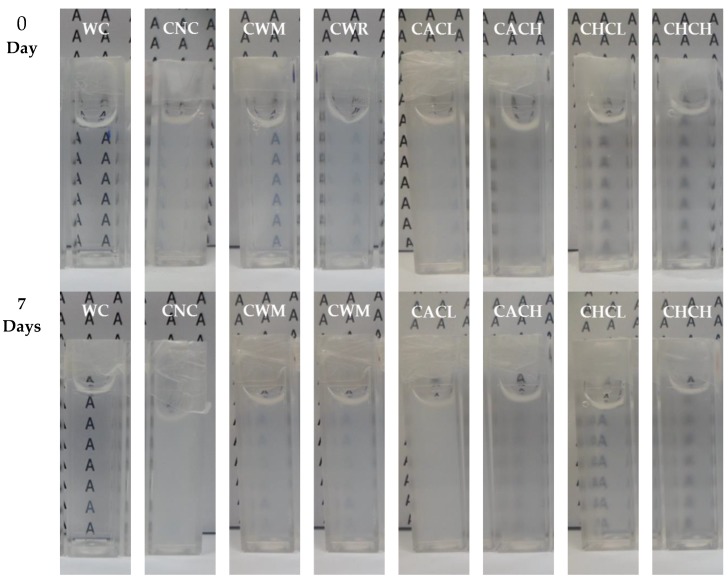
Appearance of 2% starch pastes from Thai non-GM bred waxy cassava starch (WC), commercial normal cassava starch (CNC), commercial waxy maize starch (CWM), commercial waxy rice starch (CWR) and commercial acetylated cassava starches with low and high degrees of substitutions (CACL and CACH) and commercial hydroxypropylated cassava starches with low and high degrees of molar substitutions (CHCL and CHCH) before and after storage at 4 ℃ for 7 days.

The clarity (%T) of 2% starch pastes from waxy cassava (WC) and commercial starches (CNC, CWM, CWR and stabilized starches) during storage at 4 °C for 7 days are presented in Figure 5 and their appearances before and after storage are shown in Figure 6. The paste clarity of WC, CWR and stabilized starches remained unchanged at 93%, 22% and 11%–73%, respectively, while CNC and CWM showed dramatic decreases in paste clarity after storage. It should be noted that WC exhibited the highest clarity and stability throughout the storage period, while previous research reported a lower clarity of 1% WC starch pastes [8,9] than 2% WC starch pastes analyzed in this study. 

### 2.5. Swelling Power, Solubility and Close Packing Concentration

Swelling power, solubility and close packing concentration (C*) of WC, CNC, CWM, CWR and stabilized starches are shown in Table 4. Swelling power of WC (78 g/g) was significantly higher than that of other starches (47–66 g/g), while its solubility was significantly lower (6%) than that of other starches (13%–38%). These results were in agreement with the previous findings of [11] which compared WC with NC, CWM and CWR. The solubility of WC was lower than that of CNC as there was no amylose leaching out. C* is the concentration at which the swollen granules fill up available space in starch suspension depending on temperature and is an important parameter to understand starch behavior in application [23]. The C* of WC (1%) was lower than other starches (2%–3%) indicating that WC required the lowest concentration of swollen granules to fill up starch suspension space at 85 °C in this study. 

### 2.6. Syneresis after Freeze-Thaw

**Figure 7:**
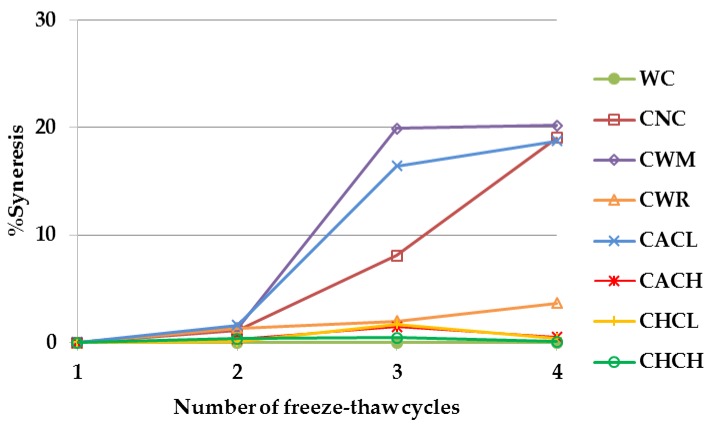
Freeze-thaw stability of 5% gels from Thai non-GM bred waxy cassava starch (WC), commercial normal cassava starch (CNC), commercial waxy maize starch (CWM), commercial waxy rice starch (CWR) and commercial acetylated cassava starches with low and high degrees of substitutions (CACL and CACH) and commercial hydroxypropylated cassava starches with low and high degrees of molar substitutions (CHCL and CHCH) during storage at –18 ℃ for 4 weeks.

Syneresis of 5% starch gels from WC, CNC, CWM, CWR and stabilized starches during storage at –18 ℃ for 4 weeks (4 freeze-thaw cycles) is illustrated in Figure 7. Only WC showed no syneresis and very stable gel during storage. Previous work by [8] also reported no syneresis of 5% starch gels from WC and CWR after storage at –20 ℃ for up to 5 weeks. Stabilized starches (except CACL) exhibited slightly higher syneresis values than WC as there were small amounts of water released from their gels during experiment.

## 3. Materials and Methods 

### 3.1. Materials

Fresh roots of 9 Thai non-GM bred waxy cassava (HB_wx_ 09-562-19, HB_wx_ 09-754-16, HB_wx_ 09-612-18, HB_wx_ 09-1041-6, HB_wx_ 09-826-2, HB_wx_ 09-317-6, HB_wx_ 09-19-2, HB_wx_ 09-635-4 and HB_wx_ 09-989-9) including three wild-type normal cassava roots (KU50, HB80 and R1) that were used as controls were obtained from the Thai Tapioca Development Institute (TTDI), Bangkok, Thailand, for starch extraction in this study. Commercial normal cassava starch (CNC) was obtained from Chorchaiwat Industry Co., Ltd. Chonburi, Thailand. Commercial waxy cereal starches including waxy maize starch (CWM) and waxy rice starch (CWR) were provided by First Starch International Co., Ltd., Bangkok, Thailand. Commercial stabilized cassava starches including acetylated cassava starch with low and high degrees of substitutions (CACL and CACH, respectively) and hydroxypropylated cassava starch with low and high degrees of molar substitutions (CHCL and CHCH, respectively) were supplied from Siam Modified Starch Corporation, Phatum Thani, Thailand. All chemical reagents used in the experiment were analytical grade.

### 3.2. Starch Isolation from Fresh Roots

Starches from fresh roots of 9 Thai non-GM bred waxy cassava varieties and 3 wild-type normal cassava varieties were extracted and purified according to [2] with modification. After washing, peeling and cutting, fresh roots were mixed in water at a ratio of 1:2 and further ground by a machine. Then, they were pressed to separate starch residue from pulp and filtered through a 90 mesh-sieve. Starch granules were precipitated for 2 h and the supernatant was decanted. Starch cake was then washed, filtered and precipitated again. Finally, starch samples were dried in a hot air oven at 50 ℃ for 16 h, ground and sieved through a 100 µm-sieve.

### 3.3. Amylose Content Determination

The analysis method for determination of amylose contents of starch samples by high performance size exclusion chromatography (HPSEC, separations module, Waters Corporation, Milford, MA, USA) was modified according to [24]. A total of 3 Ultrahydrogel HPSEC columns were connected in series. The columns including two Ultrahydrogel 120 and an Ultrahydrogel linear were maintained at 40 °C by a column oven (Shimadzu, Kyoto, Japan) and a mobile phase of deionized water was controlled at 0.8 mL min^−1^. Starch samples were gelatinized in boiling water at 0.4% (w v ^1^) for 30 min and gelatinized completely by an ultrasonic processor (Model VC 501, Sonic & Material Inc.,Newtown, CT, USA). After that, the solutions were filtered through a Millipore filter (8.0 µm) before injecting into the HPSEC system equipped with an auto-injector and refractive index detector.

### 3.4. Scanning Electron Microscopy (SEM)

Dehydrated starch samples were sprinkled on double-sided sticky tapes, mounted on circular aluminum stubs, coated with 35 nm of gold-aluminum and then observed under a Scanning Electron Microscope (SEM, FEI Quanta-450; FEI Corporation, Hillsboro, OR, USA) at an accelerating voltage of 10 kV.

### 3.5. Granule Size Distribution

Granule size distribution was determined by the method of [25]. A total of 0.75% starch suspension (w v^−1^) was prepared in 80% sucrose solution (w v^−1^). Starch granules (*n* = 500) were observed under a light microscope (Meiji Technology, Japan) and analyzed using image analysis software (Image Pro Plus 3.0, Media Cybernetic, LP).

### 3.6. Pasting Properties

Viscosity profiles of starch dispersions were analyzed with a Rapid Visco Analyzer (model RVA-4 Series, Newport Scientific, Warriewood, Australia) according to the method of [26]. Starch (1.40 g, dry basis) was dispersed in distilled water to prepare 5% starch suspension with a total weight of 28 g. Viscosity was recorded under the temperature profile: Holding at 50 ℃ for 1 min, heating from 50–95 ℃ at 12 ℃ min^−1^, holding at 95 ℃ for 2 min 30 sec, cooling down to 50 ℃ at 12 ℃ min^−1^ and then holding at 50 ℃ for 2 min with continuous stirring at 160 rpm. The following data were recorded: Peak viscosity (PV), trough viscosity (TV), breakdown (BD), final viscosity (FV), setback (SB) and pasting temperature (PT).

### 3.7. Paste Clarity

The clarity of starch paste was measured using the procedure of [27] with slight modification. A total of 2% aqueous suspensions of starches (w v^−1^) with 0.02% sodium azide (w v^−1^) were dispersed at 300 rpm for 3 min and heated in boiling water bath for 30 min with intermittent stirring. After that, the suspensions were transferred to cuvettes and cooled down at room temperature for 30 min before measuring their light transmittance (%T) at 650 nm. The starch pastes were stored at 4 ℃ and their %T were determined at every 24 h for 7 days.

### 3.8. Swelling Power, Solubility and Close Packing Concentration Measurement

Swelling power, solubility and close packing concentration (C*) were determined according to [28] with slight modifications. Starch suspensions (100 mg in 9.9 mL of deionized water) were prepared in glass tubes and sealed. After stirring at 200 rpm at room temperature for 15 min, the suspensions were heated at 85 ℃ with stirring at 200 rpm for 15 min. After cooling for 10 min at room temperature, starch suspensions were centrifuged at 2,300 rpm and 20 ℃ for 30 min. The sediments and dried supernatants were weighed and used to calculate swelling power, solubility and C* according to the following equations.

(2) $$\mathrm{Solubility}\;\left(\%\right)=\frac{dired\;supernatant\;weight\left(g\right)\times 100}{dry\;matter\;starch\;weight\left(g\right)}$$

(3) $$\mathrm{Swelling}\;\mathrm{power}\;\left(g/g\right)\;=\frac{sediment\;weight\left(g\right)\times 100}{[dry\;matter\;starch\;weight\left(g\right)\times \left(100-\%solubility\right)}$$

(4) $$\;C^{*}=\frac{dry\;matter\;starch\;weight\left(g\right)\times 100}{sediment\;weight\left(g\right)}$$

### 3.9. Freeze-Thaw Stability

To analyze starch stability after freezing and thawing, the method of [9] was applied with modification. A total of 5.0% aqueous suspensions of starches (w v^−1^) containing 0.1% sodium azide (w v^−1^) were heated in boiling water with stirring for 10 min. A total of 1.5 mL of each starch suspension was transferred to 12 microcentrifuge tubes and weighed. The starch pastes were subjected to 4 cycles of freezing at –18 ℃ for a week followed by thawing at 30 ℃ for 4 h. A total of 3 replicates of samples were taken at each cycle and centrifuged at 10,000 rpm for 10 min. The syneresis of starch paste was calculated from the amount of water released according to the following equation.

(5) $$Syneresis\left(\%\right)=\frac{weight\;of\;released\;water\times 100}{weight\;of\;starch\;paste\;before\;frozen}$$

### 3.10. Statistical Analysis

All analyses were performed in duplicate and the results were presented as mean ± SD. Significant statistical differences (*p* < 0.05) for several variables were determined using one-way ANOVA test and least significant difference (LSD) test with the SPSS program version 12.0. 

## 4. Conclusions

Thailand can successfully develop novel waxy cassava starches by a non-genetically modified (non-GM) breeding method. The granular morphology of waxy cassava starches were not different from normal cassava starches. The developed waxy cassava starches possessed better characteristics when compared with commercial waxy cereal starches (maize and rice) and commercial stabilized starches (acetylated and hydroxypropylated starches). They had a high viscosity, no retrogradation, high swelling power but low solubility, high paste clarity and stability with no syneresis after storage at –18 ℃. These waxy cassava starches indicated high potential for utilization in food industry to improve properties of products in the replacement of stabilized starches or other waxy cereal starches. Thai non-GM waxy cassava starches, however, were not tolerant to shearing and heating in processing which will be improved by cross-linking in our next research work.

## Figures and Tables

**Table 1:** Sample descriptions and amylose contents.

Sample name	Starch variety	Amylose content (%)
WC1	HB_wx_ 09-562-19 Thai non-GM bred waxy cassava	0.00 ± 0.00 ^d^
WC2	HB_wx_ 09-754-16 Thai non-GM bred waxy cassava	0.00 ± 0.00 ^d^
WC3	HB_wx_ 09-612-18 Thai non-GM bred waxy cassava	0.00 ± 0.00 ^d^
WC4	HB_wx_ 09-1041-6 Thai non-GM bred waxy cassava	0.00 ± 0.00 ^d^
WC5	HB_wx_ 09-826-2 Thai non-GM bred waxy cassava	0.00 ± 0.00 ^d^
WC6	HB_wx_ 09-317-6 Thai non-GM bred waxy cassava	0.00 ± 0.00 ^d^
WC7	HB_wx_ 09-19-2 Thai non-GM bred waxy cassava	0.00 ± 0.00 ^d^
WC8	HB_wx_ 09-635-4 Thai non-GM bred waxy cassava	0.00 ± 0.00 ^d^
WC9	HB_wx_ 09-989-9 Thai non-GM bred waxy cassava	0.00 ± 0.00 ^d^
NC1	KU50 wild-type normal cassava	18.15 ± 0.68 ^c^
NC2	HB80 wild-type normal cassava	19.59 ± 0.30 ^b^
NC3	R1 wild-type normal cassava	19.05 ± 0.68 ^b^
CNC	Commercial normal cassava	20.97 ± 0.33 ^a^
CWM	Commercial waxy maize	0.00 ± 0.00 ^d^
CWR	Commercial waxy rice	0.00 ± 0.00 ^d^

Results are mean ± S.D.; values followed by different superscripts within the same column are significantly different (*p ≤* 0.05). GM: genetic modification.

**Table 2:** Pasting properties of nine Thai non-GM bred waxy cassava starches (WC1–WC9), three wild-type normal cassava starches (NC1–NC3), commercial normal cassava starch (CNC), commercial waxy maize starch (CWM), commercial waxy rice starch (CWR) and commercial acetylated cassava starches with low and high degrees of substitutions (CACL and CACH) and commercial hydroxypropylated cassava starches with low and high degrees of molar substitutions (CHCL and CHCH).

Sample	PV (RVU)	TV (RVU)	BD (RVU)	SB (RVU)	PT (℃)
WC1	119 ± 0 ^d^	55 ± 1 ^fg^	64 ± 1 ^de^	7 ± 1 ^g^	72 ± 0 ^bcd^
WC2	122 ± 0 ^c^	56 ± 1 ^ef^	67 ± 1 ^bc^	7 ± 1 ^g^	71 ± 0 ^efgh^
WC3	126 ± 1 ^b^	58 ± 1 ^bcd^	68 ± 0 ^ab^	6 ± 1 ^g^	71 ± 0 ^fghi^
WC4	131 ± 0 ^a^	61 ± 1 ^b^	70 ± 0 ^a^	6 ± 0 ^g^	70 ± 0 ^k^
WC5	126 ± 0 ^b^	57 ± 0 ^cde^	68 ± 0 ^ab^	7 ± 1 ^g^	71 ± 0 ^hij^
WC6	116 ± 1 ^e^	56 ± 0 ^ef^	60 ± 0 ^f^	6 ± 0 ^g^	70 ± 0 ^ijk^
WC7	126 ± 1 ^b^	56 ± 0 ^def^	70 ± 1 ^a^	7 ± 0 ^g^	72 ± 1 ^bc^
WC8	120 ± 0 ^d^	58 ± 0 ^cde^	63 ± 0 ^e^	7 ± 1 ^g^	71 ± 0 ^ghi^
WC9	119 ± 1 ^d^	54 ± 1 ^g^	66 ± 0 ^cd^	10 ± 1 ^f^	72 ± 0 ^cde^
NC1	94 ± 1 ^f^	64 ± 0 ^a^	30 ± 1 ^hi^	37 ± 2 ^a^	73 ± 0 ^b^
NC2	89 ± 0 ^g^	58 ± 0 ^cde^	32 ± 0 ^gh^	31 ± 0 ^b^	71 ± 0 ^defgh^
NC3	80 ± 0 ^g^	59 ± 0 ^bc^	29 ± 0 ^i^	32 ± 1 ^b^	71 ± 0 ^cdefg^
CNC	69 ± 1 ^k^	44 ± 1 ^i^	25 ± 0 ^j^	24 ± 1 ^c^	74 ± 1 ^a^
CWM	85 ± 0 ^h^	57 ± 0 ^cde^	28 ± 0 ^i^	6 ± 0 ^g^	72 ± 0 ^cdef^
CWR	73 ± 1 ^j^	45 ± 0 ^i^	28 ± 1 ^i^	8 ± 0 ^g^	67 ± 0 ^l^
CACL	42 ± 1 ^m^	17 ± 1 ^k^	24 ± 0 ^j^	6 ± 0 ^g^	70 ± 0 ^jk^
CACH	68 ± 0 ^k^	48 ± 0 ^h^	20 ± 0 ^k^	24 ± 0 ^c^	68 ± 0 ^l^
CHCL	60 ± 1 ^l^	40 ± 1 ^j^	20 ± 0 ^k^	16 ± 2 ^e^	69 ± 1 ^l^
CHCH	79 ± 1 ^i^	46 ± 1 ^i^	33 ± 0 ^g^	20 ± 1 ^d^	64 ± 1 ^m^

Results are mean ± S.D.; values followed by different superscripts within the same column are significantly different (*p ≤* 0.05). PV: peak viscosity; TV: trough viscosity BD: breakdown viscosity; FV: final viscosity; SB: setback viscosity; PT: pasting temperature.

**Table 3:** Light transmittance at 650 nm of 2% starch pastes from WC1–WC9 and NC1–NC3 before and after storage at 4 °C for 7 days.

Sample	Light transmittance (%T) at 650 nm		Δ%T
	0 day	7 day	
WC1	94.28 ± 0.49 ^a^	94.70 ± 0.47 ^a^	0
WC2	94.80 ± 1.05 ^a^	94.83 ± 1.19 ^a^	0
WC3	93.53 ± 0.86 ^ab^	93.78 ± 0.90 ^a^	0
WC4	91.35 ± 0.56 ^b^	91.63 ± 0.15 ^a^	0
WC5	92.90 ± 2.03 ^ab^	92.93 ± 1.82 ^a^	0
WC6	93.75 ± 1.10 ^ab^	94.05 ± 1.33 ^a^	0
WC7	92.55 ± 0.99 ^ab^	92.95 ± 0.55 ^a^	0
WC8	92.45 ± 0.75 ^ab^	92.85 ± 0.87 ^a^	0
WC9	93.33 ± 0.68 ^ab^	93.95 ± 1.11 ^a^	0
NC1	56.18 ± 3.49 ^c^	36.90 ± 3.24 ^b^	34.3
NC2	53.10 ± 2.17 ^d^	27.73 ± 4.10 ^c^	47.8
NC3	53.13 ± 1.90 ^d^	29.83 ± 5.24 ^c^	43.9

Results are mean ± S.D.; values followed by different superscripts within the same column are significantly different (*p ≤* 0.05).

**Table 4:** Swelling power, solubility (%) and close packing concentration (C*) of Thai non-GM bred waxy cassava starch (WC), commercial normal cassava starch (CNC), commercial waxy maize starch (CWM), commercial waxy rice starch (CWR) and commercial acetylated cassava starches with low and high degrees of substitutions (CACL and CACH) and commercial hydroxypropylated cassava starches with low and high degrees of molar substitutions (CHCL and CHCH) determined at 85 ℃.

Sample	Swelling power (g/g)	%Solubility	C*
WC	77.65 ± 7.68 ^a^	6.40 ± 0.14 ^f^	1.38 ± 0.14 ^e^
CNC	59.44 ± 2.64 ^cd^	28.29 ± 1.67 ^b^	2.35 ± 0.05 ^bc^
CWM	50.50 ± 1.16 ^ef^	13.10 ± 0.20 ^e^	2.28 ± 0.06 ^bc^
CWR	57.56 ± 0.77 ^de^	12.64 ± 0.47 ^e^	1.99 ± 0.02 ^d^
CACL	68.43 ± 1.38 ^de^	38.06 ± 0.24 ^a^	2.36 ± 0.04 ^b^
CACH	46.77 ± 0.86 ^f^	20.16 ± 0.10 ^d^	2.68 ± 0.05 ^a^
CHCL	59.02 ± 1.06 ^d^	23.30 ± 0.24 ^c^	2.21 ± 0.03 ^c^
CHCH	66.26 ± 2.23 ^bc^	23.90 ± 1.43 ^c^	1.98 ± 0.03 ^d^

Results are mean ± S.D.; values followed by different superscripts within the same column are significantly different *(p ≤* 0.05).

## References

[1] Rolland-Sabaté A., Sánchez T., Buléon A., Colonna P., Ceballos H., Zhao S.S., Zhang P., Dufour D. (2013). Molecular and supra-molecular structure of waxy starches developed from cassava (*Manihot esculenta* Crantz). Carbohydr. Polym..

[2] Sriroth K., Santisopasri V., Pechalanuwat C., Kurotjanawong K., Piyachomkwan K., Oates C.G. (1999). Cassava starch granule structure-function properties: Influence of time and conditions at harvest on four cultivars of cassava starch. Carbohydr. Polym..

[3] Sánchez T., Salcedo E., Ceballos H., Dufour D., Mafla G., Morante N., Calle F., Pérez J.C., Debouck D., Jaramillo G. (2009). Screening of starch quality traits in cassava (*Manihot esculenta* Crantz). Starch/Stärke.

[4] John J.K., Raja K.C.M. (1999). Properties of cassava starch-dicarboxylic acid complexes. Carbohydr. Polym..

[5] Zhu F. (2015). Composition, structure, physicochemical properties, and modifications of cassava starch – Review. Carbohydr. Polym..

[6] Hsieh C.F., Liu W., Whaley J.K., Shi Y.C. (2019). Structure, properties, and potential applications of waxy tapioca starch – A review. Trends Food Sci. Technol..

[7] Bull S.E., Seung D., Chanez C., Mehta D., Kuon J.E., Truernit E., Hochmuth A., Zurkirchen I., Zeeman S.C., Gruissem W. (2018). Accelerated ex situ breeding of *GBSS*- and *PTST1*-edited cassava for modified starch. Sci. Adv..

[8] Ceballos H., Sánchez T., Morante N., Fregene M., Dufour D., Smith A.M., Denyer K., Pérez J.C., Calle F., Mestres C. (2007). Discovery of an amylose-free starch mutant in cassava (*Manihot esculenta* Crantz). J. Agric. Food Chem..

[9] Sánchez T., Dufour D., Moreno I.X., Ceballos H. (2010). Comparison of pasting and gel stabilities of waxy and normal starches from potato, maize, and rice with those of a novel waxy cassava starch under thermal, chemical, and mechanical stress. J. Agric. Food Chem..

[10] Aiemnaka P., Wongkaew A., Chanthaworn J., Nagashima S.K., Boonma S., Authapun J., Jenweerawat S., Kongsila P., Kittipadakul P., Nakasathien S. (2012). Molecular characterization of a spontaneous waxy starch mutation in cassava. Crop. Sci..

[11] Rojanaridpiched C., Vichukit V., Phumichai C. (2016). Process for improving waxy-starch cassava variety having improved qualifications and low cyanide. WO2016/118091 A2.

[12] Rolland-Sabaté A., Sánchez T., Buléon A., Colonna P., Jaillais B., Ceballos H., Dufour D. (2012). Structural characterization of novel cassava starches with low and high-amylose contents in comparison with other commercial sources. Food Hydrocoll..

[13] Karlström A., Calle F., Salazar S., Morante N., Dufour D., Ceballose H. (2016). Biological implications in cassava for the production of amylose-free starch: Impact on root yield and related traits. Front. Plant Sci..

[14] Morante N., Ceballos H., Sánchez T., Rolland-Sabaté A., Calle F., Hershey C., Gibert O., Dufour D. (2016). Discovery of new Spontaneous sources of amylose-free cassava starch and analysis of their structure and techno-functional properties. Food Hydrocoll..

[15] Pulido Diaz A., Lourdin D., Della Valle G., Fernández Quintero A., Ceballos H., Tran T., Dufour D. (2017). Thermomechanical characterization of an amylose-free starch extracted from cassava (*Manihot esculenta* Crantz). Carbohydr. Polym..

[16] Morikawa K., Nishinari K. (2000). Effects of concentration dependence of retrogradation behaviour of dispersions for native and chemically modified potato starch. Food Hydrocoll..

[17] Lawal O.S. (2004). Succinyl and acetyl starch derivatives of a hybrid maize: Physicochemical characteristics and retrogradation properties monitored by differential scanning calorimetry. Carbohydr. Res..

[18] Tran T., Piyachomkwan K., Sriroth K. (2007). Gelatinization and thermal properties of modified cassava starches. Starch/Stärke.

[19] Thomas D.J., Atwell W.A., Thomas D.J., Atwell W.A. (1988). Starch modifications. Starches.

[20] Light J.M. (1990). Modified food starches; Why, What, Where, and How. Cereal Foods World, USA.

[21] Chuenkamol B., Puttanlek C., Rungsardthong V., Uttapap D. (2007). Characterization of low-substituted hydroxypropylated canna starch. Food Hydrocoll..

[22] Jyothi A.N., Moorthy S.N., Rajasekharan K.N. (2007). Studies on the synthesis and properties of hydroxypropyl derivatives of cassava (*Manihot esculenta* Crantz) starch. J. Sci. Food Agric..

[23] Gomand S.V., Lamberts L., Visser R.G.F., Delcour J.A. (2010). Physicochemical properties of potato and cassava starches and their mutants in relation to their structural properties. Food Hydrocoll..

[24] Govindasamy S., Oates C.G., Wong H.A. (1992). Characterization of changes of sago starch components during hydrolysis by a thermostable alpha-amylase. Carbohydr. Polym..

[25] Santisopasri V., Kurotjanawong K., Chotineeranat S., Piyachomkwan K., Sriroth K., Oates C.G. (2001). Impact of water stress on yield and quality of cassava starch. Ind. Crops Prod..

[26] Newport Scientific (1995). Operation Manual of the Series 4 Rapid Visco Analyzer. Australia.

[27] Jacobson M.R., Obanni M., BeMiller J.N. (1997). Retrogradation of starches from different botanical sources. Cereal Chem..

[28] Eerlingen R.C., Jacobs H., Block K., Delcour J.A. (1997). Effects of hydrothermal treatments on the rheological properties of potato starch. Carbohydr. Res..

